# Managing Manganese: The Role of Manganese Homeostasis in Streptococcal Pathogenesis

**DOI:** 10.3389/fcell.2022.921920

**Published:** 2022-06-21

**Authors:** Shifu Aggarwal, Muthiah Kumaraswami

**Affiliations:** ^1^ Center for Molecular and Translational Human Infectious Diseases Research, Houston Methodist Research Institute, Houston, TX, United States; ^2^ Department of Pathology and Genomic Medicine, Houston Methodist Hospital, Houston, TX, United States

**Keywords:** nutritional immunity, bacterial virulence, streptococcal infection, manganese homeostasis, manganese uptake, manganese toxicity

## Abstract

Pathogenic streptococci require manganese for survival in the host. In response to invading pathogens, the host recruits nutritional immune effectors at infection sites to withhold manganese from the pathogens and control bacterial growth. The manganese scarcity impairs several streptococcal processes including oxidative stress defenses, *de novo* DNA synthesis, bacterial survival, and virulence. Emerging evidence suggests that pathogens also encounter manganese toxicity during infection and manganese excess impacts streptococcal virulence by manganese mismetallation of non-cognate molecular targets involved in bacterial antioxidant defenses and cell division. To counter host-imposed manganese stress, the streptococcal species employ a sophisticated sensory system that tightly coordinates manganese stress-specific molecular strategies to negate host induced manganese stress and proliferate in the host. Here we review the molecular details of host-streptococcal interactions in the battle for manganese during infection and the significance of streptococcal effectors involved to bacterial pathophysiology.

## Introduction

Transition metals such as iron (Fe), manganese (Mn), and zinc (Zn) are critical micronutrients required for the proliferation of pathogenic bacteria (Nairz et al., 2010; Brophy & Nolan, 2015; Lopez & Skaar, 2018; Lonergan & Skaar, 2019). Metal ions participate in major cellular processes as enzymatic or structural cofactors for various macromolecular machineries (Andreini et al., 2008). Given the near essentiality of metals for bacterial growth, the eukaryotic host evolved mechanisms to either starve or poison the invading pathogen with metals as a means to control microbial growth (Kehl-Fie & Skaar, 2010; Becker & Skaar, 2014; Makthal & Kumaraswami, 2017). As a counter mechanism, pathogenic bacteria possess molecular strategies to sense different spectrum of metal stress and overcome host nutritional defenses by employing stress-specific adaptive strategies. Thus, the battle for metals during infection is at the crossroads between health and disease and has major implications on the clinical course of infection.

Manganese plays a central role in bacterial pathogenicity due to its contribution to cellular processes that are critical for microbial growth (Bosma et al., 2021). In addition to its well-known role in bacterial antioxidant defenses, Mn also functions as a cofactor for molecules involved in carbon metabolism, nucleotide metabolism, virulence factor production, and protein synthesis (Chander et al., 1998; Boal et al., 2010; Martin & Imlay, 2011; Aguirre & Culotta, 2012; Barnese et al., 2012; Juttukonda & Skaar, 2015). As a result, the host employs intracellular and extracellular immune strategies to target bacterial Mn requirement and controls pathogen proliferation by Mn sequestration (Cellier et al., 2007; Kehl-Fie & Skaar, 2010; Brophy & Nolan, 2015). However, pathogenic bacteria evade host nutritional immune defenses by orchestrating adaptive strategies that promote its survival under Mn stress growth conditions (Kehl-Fie & Skaar, 2010; Diaz-Ochoa et al., 2014). Given the significance of Mn to bacterial pathogenesis and host defenses, elucidation of the roles of various host and bacterial molecules involved in the competition for Mn is likely to uncover new strategies to treat or prevent infections.

In this review, we discuss the molecular strategies used by streptococci to negate Mn stress during infection and their significance to bacterial virulence. Specifically, we will focus on the role of Mn homeostasis in the pathophysiology of major pathogens belong to *Streptococcus* genus that include human pathogens *S. pneumoniae*, *S. pyogenes* (known as group A *streptococcus*; GAS), *S. agalactiae* (known as group B *streptococcus*; GBS) and *S. mutans*, opportunistic pathogens *S. sanguinis* and *S. parasanguinis*, and swine pathogen *S. suis*.

## Manganese-Dependent Streptococcal Processes

Although Mn is speculated to function as a cofactor for various streptococcal proteins, only the activity of superoxide dismutases (SOD) and ribonucleotide reductases (RNR) are characterized to be Mn dependent (Makhlynets et al., 2014; Schatzman & Culotta, 2018; Ruskoski & Boal, 2021). The link between cellular Mn levels and bacterial resistance to oxidative stress is indicative of a critical role for Mn in bacterial antioxidant defenses. Superoxide dismutases are key component of streptococcal antioxidant defenses as SODs enzymatically detoxify superoxide generated by neutrophils during infection. The gene encoding *sodA* is identified in several streptococci including GAS, *S. pneumoniae, S. gordonii*, *S. agalactiae*, *S. sanguinis*, *S. mutans*, and *S. suis*, and is critical for bacterial oxidative stress resistance and virulence (Gaillot et al., 1997; Gerlach et al., 1998; Yesilkaya et al., 2000; Jakubovics et al., 2002; Tang et al., 2012; Fujishima et al., 2013; Crump et al., 2014; Turner et al., 2019). In all the characterized streptococcal SodA, Mn serves as a cofactor and SodA enzymatic activity depends on Mn uptake by Mn importers. In GAS, the activity of purified recombinant SodA is Mn dependent and SodA is inactive in its Fe-metallated or apo form (Gerlach et al., 1998). Consistent with the Mn dependency of SodA, the Mn uptake by MtsABC is critical for the elevated SodA function and the SodA activity in WT GAS increased during oxidative stress (Turner et al., 2019). Similar dependence of SodA activity on Mn uptake systems has also been observed in other streptococcal species including *S. sanguinis*, *S. pneumoniae*, and *S. gordonii* (Jakubovics et al., 2002; Crump et al., 2014; Eijkelkamp et al., 2014).

A second major cellular process affected by Mn limitation is *de novo* DNA synthesis by ribonucleotide reductases (RNR) (Boal et al., 2010; Ruskoski & Boal, 2021). The RNR catalyzes the conversion of ribonucleotides into deoxy ribonucleotides, the fundamental units required for DNA synthesis, repair, replication, and bacterial survival (Ruskoski & Boal, 2021). Typically, streptococci encode two copies of RNRs, a class III RNR that is responsible for DNA synthesis during anaerobic growth, and a class Ib RNR that catalyzes dNTP synthesis during aerobic growth (Roca et al., 2008; Makhlynets et al., 2014). In *S. sanguinis*, the catalytic activity of class Ib RNR is Mn-dependent and the Mn-metallated class Ib RNR has higher enzymatic activity than iron-bound RNRIb (Makhlynets et al., 2014). Furthermore, only the Mn-dependent class Ib RNR is required for *S. sanguinis* virulence in a rabbit model of infective endocarditis (Rhodes et al., 2014), indicating that Mn-dependent DNA synthesis by class Ib RNR is critical for *S. sanguinis* survival in the host.

## Host Mechanisms Withholding Manganese During Infection

**FIGURE 1:**
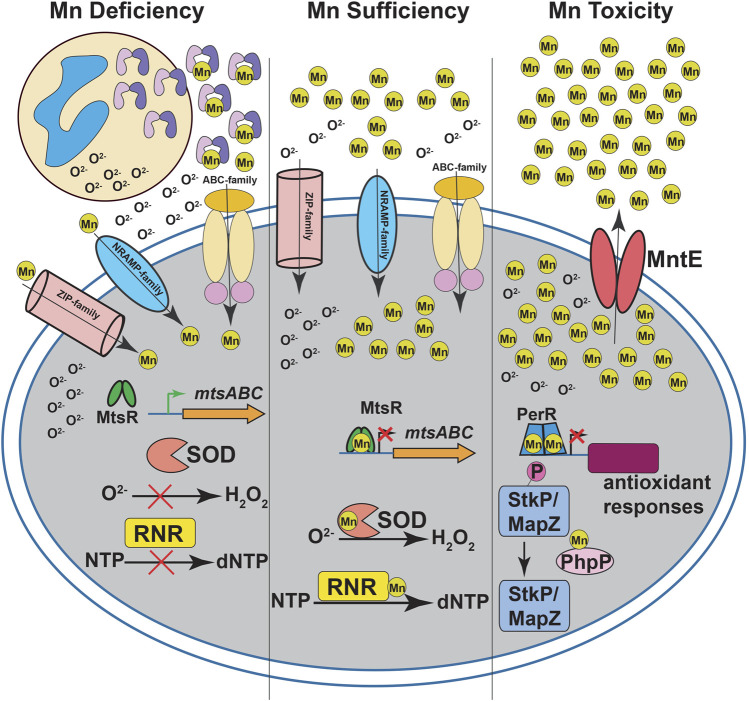
Manganese (Mn) homeostasis during streptococcal infections. During infection, neutrophils release calprotectin (CP) at infection sites (left panel). CP sequesters Mn from the invading pathogen to inhibit streptococcal growth. The intracellular Mn deficiency impairs the activity of Mn-dependent superoxide dismutases (SOD) and ribonucleotide reductases (RNR), which leads to defective antioxidant defenses against superoxide (O^2−^) and *de novo* DNA synthesis, respectively. The CP-imposed Mn deficiency is sensed by Mn-sensing metalloregulator, MtsR, and the Mn-free MtsR relieves the repression of ABC family Mn importer *mtsABC*. The Mn uptake during nutrient-limiting growth conditions in streptococci is mediated primarily by ABC transporters. In some streptococcal species, secondary transporters belonging to NRAMP- and ZIP-family transporters are also involved in Mn acquisition. During Mn sufficiency (middle panel), MtsR binds to Mn, interacts with promoters, and represses the expression of target genes including *mtsABC*. The optimal Mn levels in the cytosol promotes the activity of SOD and RNR. During Mn toxicity (right panel), the Mn efflux pump MntE detoxifies bacterial cytosol of excess Mn and promotes streptococcal survival. In the absence of MntE, high cytosolic Mn levels lead to mismetallation of peroxide sensing regulator (PerR) with Mn and results in defective antioxidant responses. Furthermore, Mn mismetallated PhpP phosphatase dephosphorylates cell division kinases StkP and MapZ, which causes improper cell division and altered bacterial cell morphologies.

The host employs extracellular as well as intracellular Mn withholding strategies to control the growth of pathogenic bacteria. The calprotectin (CP)-mediated Mn sequestration is the only characterized host mechanism that imposes extracellular Mn limitation on bacterial pathogens (Corbin et al., 2008; Kehl-Fie et al., 2011; Damo et al., 2013; Hayden et al., 2013; Diaz-Ochoa et al., 2016; Nakashige et al., 2016). CP derived from neutrophils and other host cells such as monocytes, macrophages, and epithelial cells is present in sub milligram quantities in the infected abscesses (Corbin et al., 2008; Makthal et al., 2017). The extracellular CP binds to calcium in the host tissues and chelates metals from the infection sites (Corbin et al., 2008; Hayden et al., 2013). CP, a heterodimer of S100A8 and S100A9 proteins, binds to Fe, Mn, and Zn with high affinity in calcium-dependent manner (Kehl-Fie et al., 2011; Hayden et al., 2013; Nakashige et al., 2015; Nakashige et al., 2016). The CP heterodimer has two metal binding sites located at the intersubunit interface and metal ligands for each site are provided by both S100A8 and S100A9 subunits. The His3Asp motif of site 1 constitutes the Zn-binding site and is formed by H83 and H87 of S100A8 and H20 and D30 of S100A9. The site 2 is characterized by a His6 motif that is comprised of H17 and H27 of S100A8 and H91 and H95 of S100A9 as well as H103 and H105 of the S100A9 C-terminal tail (Brophy et al., 2013; Damo et al., 2013; Nakashige et al., 2016). The site 2 with the hexa-histidine motif can bind Mn, Fe, and Zn *in vitro* (Brophy et al., 2013; Nakashige et al., 2015; Nakashige et al., 2016; Zygiel & Nolan, 2018). The effect of Mn sequestration by CP on bacterial pathogenesis has been well characterized in *S. aureus* and gram-negative bacteria (Kehl-Fie et al., 2011; Kehl-Fie et al., 2013; Diaz-Ochoa et al., 2016). The CP-imposed Mn deficiency impairs the enzymatic detoxification of reactive oxygen species (ROS) by Mn-dependent superoxide dismutase (SOD) in *S. aureus*, which leads to increased susceptibility to oxidative stress, reduced bacterial survival, and attenuated bacterial virulence (Kehl-Fie et al., 2011; Kehl-Fie et al., 2013) (Figure 1). CP is present in GAS-infected abscesses (Makthal et al., 2017), and CP-mediated Zn sequestration impacts streptococcal growth and pathogenesis (De Filippo et al., 2014; Makthal et al., 2017; Burcham et al., 2020; Makthal et al., 2020). However, the significance of Mn withholding by CP on streptococcal proliferation *in vivo* and virulence remains unelucidated. Analyses of the growth kinetics of *∆sloC* and ∆*mntH* mutants of *S. mutans* in the presence of CP *in vitro* indicated that Mn import by SloC and MntH is critical for *S. mutans* CP resistance (Kajfasz et al., 2020). Thus, as observed in other pathogens, it is likely that streptococcal pathogens may also engage in competition for Mn with CP during infection.

In addition to extracellular Mn chelation, the host also limits Mn availability to intracellular bacteria by employing natural resistance-associated macrophage protein 1 (NRAMP1) transporters (Jabado et al., 2000; Zaharik et al., 2004; Peracino et al., 2006; Cellier et al., 2007). The NRAMP1 importers are associated with the lysosomes and proposed to transport Mn and Fe out of the phagolysosomes into host cytosol (Jabado et al., 2000; Cellier et al., 2007). The Mn withdrawal by NRAMP1 from phagolysosomes is suggested to starve the phagocytosed pathogen of Mn and impair bacterial Mn-dependent cellular processes including antioxidant defenses and bacterial survival. Consistent with this, NRAMP1 plays a major role in controlling the pathogenicity of *Salmonella enterica* in mouse models of infection (Zaharik et al., 2004). Experimental evidence for the role of NRAMP1 in host defenses against streptococcal growth is lacking and additional investigations are required to assess the contribution of NRAMP1 in limiting intracellular streptococcal growth.

## Streptococcal Strategies to Acquire Manganese

Despite the presence of host immune mechanisms withholding Mn, streptococcal pathogens colonize the host and establish infection, suggesting that pathogens possess mechanisms to acquire Mn and evade host-imposed Mn sequestration. The streptococcal species deploy three different family of Mn acquisition systems to obtain extracellular Mn. These transporters belong to ATP-binding cassette (ABC)-family, natural resistance-associated macrophage protein (NRAMP) family, and ZRT-, IRT-like protein (ZIP) family of transporters (Figure 1). The ABC-family manganese transporters are universally present in all the characterized streptococcal genomes (Burnette-Curley et al., 1995; Kolenbrander et al., 1998; Kitten et al., 2000; Janulczyk et al., 2003; Paik et al., 2003; Johnston et al., 2004; Schreur et al., 2011; Crump et al., 2014; Kajfasz et al., 2020). Contrary to this, the NRAMP-family Mn transporters are less abundant among streptococcal species (Bray et al., 2009; Shabayek et al., 2016; Kajfasz et al., 2020), whereas a ZIP family Mn importer is characterized only in *S. sanguinis* (Puccio et al., 2022a).

The ABC family of transporters are the primary streptococcal Mn importers and the best studied. All the characterized ABC-family Mn importers share similar molecular architecture and are composed of three components: a surface-exposed, membrane anchored solute binding protein that binds to extracellular Mn with high affinity, an integral membrane protein that mediates Mn influx, and a cytoplasmic ATPase that facilitates Mn import by ATP hydrolysis (Li et al., 2014; Neville et al., 2021). The well characterized ABC transporters that are involved in streptococcal Mn acquisition include PsaBCA of *S. pneumoniae*, MtsABC of *S. pyogenes*, ScaCBA of *S. gordonii*, SloABC of *S. mutans*, TroABC of *S. suis*, SsaACB of *S. sanguinis*, and FimABC of *S. parasanguinis* (Burnette-Curley et al., 1995; Berry & Paton, 1996; Kolenbrander et al., 1998; Kitten et al., 2000; Janulczyk et al., 2003; Paik et al., 2003; Johnston et al., 2004; Schreur et al., 2011; Crump et al., 2014; Kajfasz et al., 2020)*.* Since streptococci also use ABC transporters for the import of Fe and Zn (Sun et al., 2010; Makthal et al., 2017; Makthal & Kumaraswami, 2017), it is challenging to assign the cognate ligand for these transporters. A parallel approach that includes the identification of Mn-specific binding motifs in the solute-binding subunit of ABC transporters along with the characterization of importer-inactivated mutants for Mn-specific biological phenotypes is often required to identify the cognate ligand for ABC transporters. Most of the well characterized Mn-binding extracellular subunits of streptococcal ABC transporters such as PsaA from *S. pneumoniae*, MtsA from GAS, ScaA from *S. gordonii*, SloC from *S. mutans*, and FimA from *S. parasanguinis* contain a His-His-Glu-Asp motif that binds Mn (Sun et al., 2009; Couñago et al., 2014). Contrary to this, the Zn-binding motif in the surface-exposed Zn-binding subunit AdcA of streptococcal Zn importers AdcABC contain a His-His-His-Glu motif (Luo et al., 2021). However, the *S. suis* Mn importer TroA has a Zn-like His-His-His-Asp binding motif, which underscores the necessity to define the metal-specific biological phenotypes to assign the correct ligand for ABC-family metal importers (Schreur et al., 2011). Some of the biological phenotypes to identify Mn-specific transporters include Mn-specific transcription regulation of the genes encoding transporters, and phenotypes associated with transporter inactivated mutants such as reduced intracellular Mn levels, defective growth in Mn limiting growth conditions, restoration of growth specifically by Mn supplementation, and increased sensitivity to oxidative stress. In accordance with this, inactivation of *psaABC* from *S. pneumoniae*, *mtsABC* from GAS, *scaABC* from *S. gordonii*, *ssaABC* from *S. sanguinis*, *fimABC* from *S. parasanguinis,* and *troABC* from *S. suis* resulted in decreased cytosolic Mn levels, impaired growth during Mn limitation, restoration of growth by exogenous provision of Mn, and defective antioxidant defenses (Burnette-Curley et al., 1995; Kolenbrander et al., 1998; Kitten et al., 2000; Janulczyk et al., 2003; Paik et al., 2003; Johnston et al., 2004; Schreur et al., 2011; Crump et al., 2014; Kajfasz et al., 2020).

*S. agalactiae* and *S. mutans* employ two Mn transporters to cope with Mn limitation. The Mn uptake in *S. mutans* is mediated by an ABC-transporter *sloABC* and a NRAMP-family importer *mntH* (Kajfasz et al., 2020). The single ∆*sloC* or ∆*mntH* mutant had WT-like phenotype in Mn uptake, growth in Mn limiting growth conditions, and oxidative stress tolerance (Kajfasz et al., 2020). However, inactivation of both transporters resulted in defective phenotypes typically associated with Mn deficiency *in vitro* (Kajfasz et al., 2020), indicating the functional redundancy and complementarity between the two importers for Mn acquisition by *S. mutans*. Intriguingly, when tested for their resistance against CP *in vitro*, the ∆*sloC* mutant was more sensitive to CP, whereas the ∆*mntH* mutant had WT-like resistance to CP, suggesting that the two transporters may have distinct roles in *S. mutans* resistance to CP-mediated metal limitation (Kajfasz et al., 2020). Although two different Mn transporters are implicated in Mn uptake by *S. agalactiae*, their collective roles in Mn acquisition are not fully defined. *S. agalactiae* encodes an ABC-transporter *mtsABC* and a NRAMP-family importer *mntH* (Bray et al., 2009; Shabayek et al., 2016; Zhu et al., 2020). Consistent with a role in Mn acquisition, the *mtsA* expression is upregulated during Mn limitation (Bray et al., 2009) and *mtsABC* is critical for oxidative stress resistance and *S. agalactiae* survival in human blood and plasma (Zhu et al., 2020). Similarly, the *mntH* is also involved in Mn uptake and antioxidant defenses of *S. agalactiae* (Shabayek et al., 2016). Further studies are required to delineate the individual as well as collective roles of the two Mn transporters in Mn acquisition, antioxidant defenses, CP resistance, and virulence of *S. agalactiae*.

*S. sanguinis* encode three Mn transporters, ABC-transporter SsaACB, NRAMP-transporter MntH, and ZIP family transporter TmpA (Puccio et al., 2022b). Functional characterization of the transporters indicate that SsaACB is the primary Mn transporter, whereas TmpA and MntH are functionally redundant secondary transporters. Inactivation of either *tmpA* or *mntH* alone did not cause any Mn-associated defective phenotypes. However, the double mutant *∆ssaACB∆tmpA* was more defective than the single ∆*ssaABC* mutant in Mn acquisition, and survival in Mn limiting growth conditions (Puccio et al., 2022a). The growth phenotype of triple mutant *∆ssaACB∆tmpA∆mntH* in Mn limiting growth conditions was similar to the double mutant, suggesting the functional redundancy of secondary transporters (Puccio et al., 2022b).

## Manganese Acquisition and Streptococcal Virulence

Consistent with the significant contribution of Mn importers for streptococcal growth in the presence of various stressors *in vitro*, the Mn uptake systems are critical for streptococcal virulence in several animal models of systemic infection mimicking various forms of streptococcal disease manifestations. The *psaABC* in *S. pneumoniae*, and *troA* in *S. suis* are critical for bacterial virulence in intraperitoneal or intravenous mouse models of systemic infection (Marra et al., 2002; Johnston et al., 2004; McAllister et al., 2004; Schreur et al., 2011). Similarly, the bacterial virulence of ∆*sloC* or ∆s*loA* of *S. mutans*, ∆*fimA* of *S. parasanguinis*, and ∆*ssaABC* of *S. sanguinis* was significantly attenuated in animal models of infective endocarditis (Burnette-Curley et al., 1995; Kitten et al., 2000; Paik et al., 2003; Crump et al., 2014). Intriguingly, the deletion of secondary Mn transporters *mntH* or *tmpA* alone did not affect the virulence phenotype *S. sanguinis* in rabbit model of infective endocarditis (Puccio et al., 2022a). However, deletion of either *mntH* or *tmpA* in ∆*ssaABC* mutant strain caused further reduction in bacterial survival *in vivo* compared to single ∆*ssaABC* mutant (Puccio et al., 2022a). These results suggest that the ABC-transporter SsaABC is the primary Mn uptake system *in vivo*, however, MntH or TmpA also has a role, albeit a lesser role, in Mn acquisition during *S. sanguinis* infection. In GAS, the Mn import by MtsABC contributes significantly to bacterial pathogenesis in a subcutaneous air sac model of invasive infection as inactivation of *mtsABC* resulted in reduced mortality compared to wild type GAS (Janulczyk et al., 2003). Contrary to the systemic mouse models of infection, the Mn importer mutant strains had a varied virulence phenotype in experimental models simulating localized streptococcal infections. In *S. mutans*, inactivation of either ABC-transporter *sloC* or NRAMP-transporter *mntH* failed to affect bacterial survival *ex vivo* in human saliva (Kajfasz et al., 2020). However, when both transporters were inactivated, the ∆*sloC∆mntH* double mutant strain had defective growth in saliva (Kajfasz et al., 2020). Similarly, inactivation of *sloC* failed to affect *S. mutans* plaque formation in gnotobiotic rat model of caries (Kitten et al., 2000). The *S. sanguinis* Mn importer SsaABC is dispensable for bacterial colonization in a mouse model of oral colonization (Puccio et al., 2022b). Contrary to this, the Mn importer PsaABC is critical for of S*. pneumoniae* colonization in mouse model of intranasal colonization. Inactivation of different components of *psaABC* system caused decreased nasopharyngeal colonization, reduced bacterial burden in lungs, and decreased mortality (Berry & Paton, 1996; Marra et al., 2002; McAllister et al., 2004). These observations suggest that pathogen-specific and host niche-specific variations exist in the contribution of streptococcal Mn importers to bacterial pathogenesis.

Despite the demonstrated significance of Mn importers to streptococcal virulence, their roles in direct competition with host Mn sequestration systems such as calprotectin *in vivo* are yet to be demonstrated. Future investigations assessing the contribution of Mn uptake systems to streptococcal pathogenesis in mice lacking CP or NRAMP1 transporters are required to delineate the precise role of Mn importers in streptococcal resistance to host-imposed Mn limitation.

## Manganese Toxicity and Streptococcal Pathogenesis

Unlike CP-mediated Mn limitation on the pathogen, evidence for host mechanisms imposing Mn toxicity is lacking. However, the ubiquitous presence of genes encoding Mn exporters in streptococcal genomes and their demonstrated significance to bacterial pathogenesis in animal models of infection suggest that pathogens encounter Mn toxicity *in vivo*. The Mn efflux pump MntE has been identified and characterized in *S. pneumoniae*, GAS, *S. mutans*, and *S. suis* (Rosch et al., 2009; Turner et al., 2015; Xu et al., 2017; O'Brien et al., 2020) (Figure 1). MntE belongs to cation diffusion facilitator (CDF) family of transporters that couple import of H^+^ ions with Mn efflux (Kolaj-Robin et al., 2015; Martin & Giedroc, 2016). The MntE exporters exist as a dimer and each subunit is composed of an amino terminal transmembrane domain (TMD) with 6 transmembrane helices and a carboxy terminal cytoplasmic domain (CTD). Both domains are critical for Mn export by MntE (Martin & Giedroc, 2016). Although 3 metal binding sites, sites A-C, are identified, the significance of each site for metal binding and metal export vary among CDF transporters. The metal binding site A located in the transmembrane domain constitutes the active site for metal transport and has the determinants for metal selectivity (Martin & Giedroc, 2016). In MntE and its paralogs, the site A is composed of a Asn-Asp-Asp-Asp (N-D-D-D) motif with contributions from amino acids Asn47 and Asp51 of transmembrane helix TM2 and Asp155 and Asp159 of TM5. Inactivating alanine substitutions at each of the site 1 amino acids of pneumococcal MntE resulted in increased Mn sensitivity, indicating the critical role of site 1 in Mn efflux by MntE (Martin & Giedroc, 2016). Comparison of the amino acid sequence of MntE with Zn-binding CDF exporters identified a H-D-H-D motif in Zn exporters in place of N-D-D-D motif of MntE. Interestingly, swapping studies substituting N-D-D-D motif of MntE with H-D-H-D motif switched the metal specificity and export activities of MntE from Mn to Zn, suggesting that site A of MntE contains the specificity determinant for Mn binding and efflux (Martin & Giedroc, 2016). The site B is located at the interdomain interface between TMD and CTD and involved in metal binding. However, it remains unclear whether metal binding at site B participates in Mn influx directly or indirectly by influencing MntE stability and/or Mn binding at site A (Martin & Giedroc, 2016). The site C is critical for metal efflux by Zn exporters, however, it is dispensable for Mn efflux by MntE (Martin & Giedroc, 2016).

Although the full extent of cellular processes affected by Mn toxicity is yet to be elucidated, few streptococcal pathophysiological mechanisms influenced by Mn excess have been characterized. Invariably, the mismetallation of non-cognate molecular targets with Mn during Mn excess is the basis for the observed defective phenotypes. GAS upregulate *mntE* expression selectively in response to Mn excess (Turner et al., 2015), suggesting that GAS deploy MntE to negate Mn toxicity. Inactivation of *mntE* resulted in increased intracellular accumulation of Mn, impaired oxidative stress defenses, and growth defect during GAS growth *in vitro* in the presence of excess Mn (Turner et al., 2015). Further characterization of MntE suggested that mismetallation of GAS peroxide stress sensor PerR during Mn toxicity is likely the basis for the impaired GAS antioxidant defenses (Figure 1). The Mn- or Fe-metallated PerR represses the transcription of genes involved in GAS oxidative stress defenses. During oxidative stress, only the Fe-bound PerR is responsive to reactive oxygen species and responds by derepressing GAS oxidative stress regulon (Makthal et al., 2013). Contrary to this, the PerR:Mn is recalcitrant to oxidative stress and metallation with Mn during Mn surplus locks PerR in repressor state. Consequently, the failure of Mn mismetallated PerR to derepress oxidative stress regulon in response to ROS likely contributes to impaired GAS antioxidant defenses (Makthal et al., 2013). Consistent with this, unlike the increased sensitivity of ∆*mntE* mutant to oxidative stress, the ∆*perR∆mntE* double mutant had WT-like resistance to peroxide stress (Turner et al., 2015). Similarly, the Mn-metallated PerR in ∆*mntE* mutant was proposed to cause repression of a secreted Dnase *sdaI*, a major GAS virulence determinant that is critical for the degradation of neutrophil extracellular traps (NETs) (Grifantini et al., 2011; Wang et al., 2013). Consequently, the impaired antioxidant defenses of ∆*mntE* mutant caused defective GAS survival during growth in the presence of neutrophils (Turner et al., 2015). In pneumococci, intracellular Mn excess caused by the inactivation of *mntE* resulted in asymmetric cell division and cells with altered morphologies. During Mn toxicity, the ratio of Mn:Zn was altered, which resulted in Mn mismetallation and hyperactivity of a cell division regulator, PhpP phosphatase (Martin et al., 2017). The increased phosphatase activity of PhpP led to dephosphorylation of cell division regulatory kinases, StkP and MapZ, and likely contribute to the dysregulation of pneumococcal cell division (Figure 1) (Martin et al., 2017). Transcriptome analyses of pneumococcal ∆*mntE* mutant revealed that genes encoding pilus regulator *rlrA* and pilus subunit *rrgB* were upregulated during Mn excess (Rosch et al., 2009). However, the downstream consequences of pilus upregulation during Mn intoxication to pneumococcal pathophysiology remain unclear.

The assessment of ∆*mntE* virulence phenotype in animal models of infection revealed niche-specific roles for Mn exporters in streptococcal pathogenesis. The pneumococcal ∆*mntE* mutant was attenuated for nasal colonization, dissemination to secondary sites, and bacterial survival in intranasal mouse model of pneumococcal infection (Rosch et al., 2009). Similarly, competition infection studies with *S. suis* WT and *∆mntE* mutant showed that the ∆*mntE* mutant had reduced survival compared to WT in systemic mouse model of infection (Xu et al., 2017). Contrary to this, inactivation of *mntE* did not affect GAS virulence in transgenic humanized plasminogen mouse model of invasive infection (Turner et al., 2015). These observations suggest that host-imposed Mn intoxication is an anatomic site-specific nutritional immune mechanism and MntE is critical for streptococcal virulence in specific host niches.

## Manganese Sensing and Gene Regulation During Manganese Limitation by Streptococci

Pathogens are tasked with the challenge of sensing host induced alterations in Mn levels and maintaining the balance between acquiring sufficient Mn and not incurring Mn toxicity during infection. Successful pathogens monitor the fluctuations in Mn levels via cytosolic Mn sensing transcription regulators and tightly regulate the Mn stress-specific adaptive responses including Mn uptake (Merchant & Spatafora, 2014; Chandrangsu et al., 2017). During Mn sufficiency, Mn-sensing transcription regulators bind Mn, interact with target promoters with high affinity, repress expression of Mn importers, and reduce Mn uptake (Figure 1). Conversely, under Mn limiting conditions, the metal-free apo transcription regulators dissociate from the promoters, induce gene expression by relieving the repression, and facilitate Mn acquisition *via* upregulation of Mn importers (Figure 1) (Merchant & Spatafora, 2014; Do et al., 2019). The streptococcal Mn sensors belong to a highly conserved diphtheria toxin repressor (DtxR)-family of transcription regulators that include PsaR from *S. pneumoniae*, MtsR from GAS, SloR from *S. mutans*, ScaR from *S. gordonii*, and TroR from *S. suis* (Jakubovics et al., 2000; Paik et al., 2003; Johnston et al., 2006; Stoll et al., 2009; Do et al., 2019; Zheng et al., 2021).

Structural studies of MtsR and ScaR dimers revealed that these regulators adopt a typical DtxR family fold with 3 distinct domains per subunit: an amino-terminal DNA-binding domain with a winged helix-turn-helix (wHTH) DNA binding motif, a central dimerization domain, and a carboxy-terminal FeoA-like domain (Stoll et al., 2009; Do et al., 2019). Each MtsR subunit has two metal sensing sites that are located in the central dimerization domain. The Mn sensing site 1 is at the interface between the dimerization and C-terminal FeoA domains, whereas the site 2 is situated between the dimerization and N-terminal DNA-binding domains. The MtsR amino acids His76, Glu80, Cys123, and His125 constitute Mn site 1, whereas the site 2 is made of His32, His95, His161, and Asp163 (Do et al., 2019). Metal occupancy in MtsR promotes high affinity interactions between MtsR and *mts* motifs in target promoters and mediates Mn-dependent transcription repression by MtsR. Both sites are critical for metalloregulation by MtsR and single alanine substitutions at His76 and E80 of the site 1 as well as His95 and His161 of the site 2 caused reduced Mn binding, decreased affinity of MtsR for *mts* motifs, defective promoter binding by MtsR, and loss of transcription repression of target genes in the presence of Mn. Although both sites are critical for metal binding and gene regulation by MtsR, the disruption of site 1 had greater effect than site 2 with the site 1 mutants had complete loss of repression, while the site 2 mutants exhibited only partial loss of repression (Do et al., 2019). These observations prompted the speculation that the site 1 is the primary Mn sensing site and the site 2 is the secondary Mn sensing site, and the dissociation of Mn from these sites may allow gradual derepression of target genes in response to the degree of severity of Mn deficiency. The site 1 metal ligands are conserved among all the characterized streptococcal Mn sensing regulators and the site 1 ligands His76, Glu80, Cys123, and His125 in SloR are critical for promoter binding and transcription regulation (Haswell et al., 2013). As observed in MtsR, the site 2 in SloR is also located between the dimerization and N-terminal DNA-binding domains (Haswell et al., 2013). However, the SloR site 2 is distinct from that of MtsR as it varies in the amino acid composition (Do et al., 2019). The site 2 in SloR is composed of Asp7, Glu99, Glu102, and His103 and the site 2 metal ligands are critical for SloR metalloregulation (Haswell et al., 2013). Thus, the metal sensing and gene regulation by site 1 is likely common among all the streptococcal Mn metalloregulators and the composition of site 2 may vary.

Although MtsR exists as a dimer in solution, MtsR oligomerizes upon binding to the promoter. The structural analyses of MtsR uncovered an intermolecular interface between the C-terminal FeoA domains of adjacent MtsR dimers that is involved in MtsR oligomerization (Do et al., 2019). Consistent with this observation, alanine substitutions in the oligomerization interface did not affect MtsR dimerization, however, it disrupted MtsR multimerization on target promoter and caused loss of repression of target gene expression by MtsR (Do et al., 2019). The oligomerization of Mn sensors on promoter sequences has also been observed for SloR from *S. mutans* and ScaR from S*. gordonii* (Jakubovics et al., 2000; Spatafora et al., 2015) and the amino acids involved in MtsR oligomerization are conserved among Mn sensing streptococcal transcription regulators. Further characterization of the C-terminal FeoA domain-mediated oligomerization of other streptococcal Mn sensors on the target promoters and its impact on their gene regulation is required to assess whether FeoA domain has a similar role in other Mn-sensing metalloregulators.

Comparative transcriptome analyses of several streptococci with their corresponding Mn-sensing regulator inactivated strains provided critical insights into the streptococcal adaptive responses that aid bacterial growth during Mn scarcity. In most cases, the Mn-sensing regulators primarily control the expression of streptococcal genes encoding ABC-family Mn importers in response to Mn availability. Three independent studies assessing the regulatory influence of PsaR and Mn showed that the core components of *S. pneumoniae* adaptive responses to Mn limitation include Mn importer *psaABC*, the choline binding virulence factor *pcpA*, and secreted serine protease *prtA* (Johnston et al., 2006; Hendriksen et al., 2009; Ogunniyi et al., 2010). However, strain-specific variations were observed in PsaR regulon as inactivation of *psaR* in *S. pneumoniae* strain D39 resulted in 37 differentially regulated genes, whereas the expression of 19 genes were altered in ∆*psaR* mutant in *S. pneumoniae* TIGR4 background (Hendriksen et al., 2009). Although a larger GAS MtsR regulon was previously reported (Toukoki et al., 2010), a recent study showed that the core MtsR regulon is much smaller and consists of 26 genes (Do et al., 2019). The MtsR-regulated GAS adaptive responses to Mn limitation include Mn importer *mtsABC*, *sia* operon encoding putative iron transport system, iron efflux pump *pmtA*, and ribonucleotide reductases (Do et al., 2019). Similarly, transcription profiling of ∆*troR* mutant in *S. suis* revealed that TroR primarily controls the expression of Mn importer *troABCD* in response to Mn limitation (Zheng et al., 2021). Contrary to these observations, SloR in *S. mutans* exerts major regulatory influence with a total of 686 genes are differentially regulated in the ∆*sloR* mutant compared to WT (O'Rourke et al., 2010). In addition to Mn importer *sloABC*, some of the characterized SloR-repressed genes include *pdhABC* genes involved in sugar and amino acid metabolism, *citBZC* genes encoding citrate synthase, and *glgBCDA* operon involved in carbohydrate metabolism. Interestingly, SloR also functions as a Mn-dependent activator and induces the expression of genes encoding thiol peroxidase *tpx*, and bacteriocin transporter complex *bta* (O'Rourke et al., 2010).

An efficient adaptation to fluctuating Mn levels during infection is likely critical for bacterial virulence. Thus, Mn sensing and regulation of streptococcal adaptive responses by metalloregulators can be critical for bacterial virulence as dysregulation of Mn uptake may cause intracellular Mn stress. Consistent with this, Consistent with this, the Mn sensing, oligomerization, and gene regulation function of MtsR is cricital for GAS virulence in an intramuscular mouse model of invasive infection (Do et al., 2019). Contrary to this, gene regulation by TroR is dispensable for *S. suis* virulence in an intraperitoneal mouse model of infection (Zheng et al., 2021). Similarly, inactivation of *psaR* failed to affect *S. pneumoniae* colonization in a mouse model of nasopharyngeal colonization. However, the ∆*psaR* mutant had minimal but inconsistent differences in virulence in mouse models of pneumonia compared to their parental strain (Hendriksen et al., 2009). These observations suggest that Mn availability and the requirement for tight regulation of Mn uptake may vary in different host niches.

## Summary and Future Perspectives

Growing evidence suggest that Mn has a critical role in various cellular processes associated with the pathophysiology of streptococci during infection. However, with the exception for few examples, the full repertoire of Mn-dependent streptococcal molecular machineries and cellular processes remains unknown. Efforts are required to identify bacterial processes that require Mn as such evidence will not only identify new Mn-dependent bacterial proteins and processes but also uncover additional evasive strategies used by pathogens to overcome host nutritional immune defenses.

It is evident that streptococcal pathogens experience host induced Mn stress during infection. However, little is known about the host factors involved, and the nature and spatiotemporal distribution of Mn based nutritional immune mechanisms employed by the host during streptococcal infections. Given the significant differences in Mn levels among various host tissues (Aschner & Aschner, 2005; Kehl-Fie et al., 2013; Juttukonda et al., 2017; Monteith et al., 2022) and Mn requirements among different pathogens (Chandrangsu et al., 2017), it is critical to determine the nature of host nutritional immune strategies at different host sites and assess their contribution to the prevention of infections caused by various streptococcal pathogens. Elucidation of the host site-specific and streptococcal species-specific molecules involved in the host-pathogen competition for Mn may improve our understanding of bacterial pathogenesis and pave the foundation for developing novel therapeutic strategies.
